# Supplementary material for: Evaluating the effect of stand properties and site conditions on the forest reflectance from Sentinel-2 time series
Source: PLoS One. 2021 Mar 15;16(3):e0248459. doi: 10.1371/journal.pone.0248459 (PMC7959393; doi:10.1371/journal.pone.0248459)
Supplement: S1 Table — (DOCX) [file pone.0248459.s001.docx]

**S1 Table. Adjusted R^2^ values for the GAMs regression considering the elevation and reflectance of common beech stands during selected dates.**

|  | Blue | Green | Red | RE1 | RE2 | RE3 | NIR1 | NIR2 | SWIR1 | SWIR2 |
| --- | --- | --- | --- | --- | --- | --- | --- | --- | --- | --- |
| April 15th | 0.07 | 0.12 | 0.06 | 0.05 | 0.13 | 0.10 | 0.10 | 0.08 | 0.02 | 0.03 |
| April 20th | 0.08 | 0.19 | 0.08 | 0.05 | 0.26 | 0.24 | 0.19 | 0.19 | 0.03 | 0.07 |
| May 2nd | **0.57** | 0.08 | 0.38 | 0.13 | **0.43** | **0.49** | **0.50** | **0.47** | 0.10 | 0.19 |
| May 7th | 0.21 | 0.30 | 0.29 | 0.37 | 0.27 | 0.38 | 0.38 | 0.39 | 0.11 | 0.15 |
| May 12th | 0.17 | **0.40** | 0.24 | **0.44** | **0.04** | 0.13 | 0.14 | 0.11 | 0.06 | 0.13 |
| Oct 14th | 0.02 | 0.28 | 0.07 | 0.14 | 0.35 | 0.35 | 0.27 | 0.27 | 0.12 | 0.28 |
| Oct17th | 0.06 | 0.33 | 0.18 | **0.43** | **0.52** | **0.53** | **0.55** | **0.46** | 0.01 | 0.04 |
| Oct 24th | 0.13 | 0.26 | 0.06 | 0.11 | 0.16 | 0.14 | 0.11 | 0.07 | 0.01 | 0.01 |
| Oct 27th | 0.15 | 0.05 | 0.03 | 0.05 | 0.15 | 0.13 | 0.12 | 0.05 | 0.02 | 0.02 |
