# Supplementary material for: Evaluating the effect of stand properties and site conditions on the forest reflectance from Sentinel-2 time series
Source: PLoS One. 2021 Mar 15;16(3):e0248459. doi: 10.1371/journal.pone.0248459 (PMC7959393; doi:10.1371/journal.pone.0248459)
Supplement: S2 Table — (DOCX) [file pone.0248459.s002.docx]

**S2 Table. Adjusted R^2^ values for the GAMs regression considering the age and reflectance of Silver fir stands during selected dates (>=80% same-age-dominated fir stands).**

| Date | Blue | Green | Red | RE1 | RE2 | RE3 | NIR1 | NIR2 | SWIR1 | SWIR2 |
| --- | --- | --- | --- | --- | --- | --- | --- | --- | --- | --- |
| April 15^th^ | 0.20 | 0.26 | 0.25 | 0.26 | **0.56** | **0.56** | **0.57** | **0.46** | 0.31 | 0.29 |
| April 20^th^ | 0.21 | 0.31 | 0.25 | **0.40** | **0.55** | **0.56** | **0.58** | **0.40** | 0.32 | 0.30 |
| May 2^nd^ | 0.22 | 0.20 | 0.20 | 0.32 | 0.31 | 0.32 | 0.32 | 0.25 | 0.30 | 0.28 |
| May 7^th^ | 0.30 | **0.43** | 0.34 | **0.46** | 0.39 | 0.38 | 0.28 | 0.29 | 0.37 | 0.34 |
| May 12^th^ | 0.31 | **0.50** | 0.27 | **0.47** | **0.43** | **0.41** | **0.41** | 0.33 | 0.35 | 0.32 |
| June 16^th^ | 0.16 | 0.31 | 0.18 | 0.39 | **0.42** | 0.39 | 0.37 | 0.32 | 0.24 | 0.17 |
| July 1^st^ | 0.07 | 0.33 | 0.10 | **0.42** | **0.48** | **0.47** | **0.48** | 0.37 | 0.31 | 0.25 |
| August 28^th^ | 0.04 | 0.17 | 0.13 | 0.32 | **0.46** | **0.46** | **0.50** | 0.39 | 0.30 | 0.24 |
| Sep 22th | 0.29 | 0.35 | 0.33 | **0.41** | **0.53** | **0.53** | **0.52** | **0.46** | 0.33 | 0.31 |
| October 2^nd^ | 0.03 | 0.08 | 0.08 | 0.21 | 0.38 | **0.40** | 0.39 | 0.27 | 0.28 | 0.20 |
| October 14^th^ | 0.30 | 0.29 | 0.30 | **0.41** | **0.48** | **0.50** | **0.51** | **0.45** | 0.35 | 0.34 |
| Oct17^th^ | 0.08 | 0.17 | 0.17 | 0.31 | **0.43** | **0.47** | **0.47** | 0.39 | 0.28 | 0.25 |
| Oct 24^th^ | 0.01 | 0.06 | 0.07 | 0.17 | 0.29 | 0.32 | 0.33 | 0.19 | 0.26 | 0.21 |
| Oct 27^th^ | 0.22 | 0.31 | 0.29 | **0.40** | **0.49** | **0.49** | **0.49** | **0.51** | 0.33 | 0.30 |
