# Supplementary material for: Evaluating the effect of stand properties and site conditions on the forest reflectance from Sentinel-2 time series
Source: PLoS One. 2021 Mar 15;16(3):e0248459. doi: 10.1371/journal.pone.0248459 (PMC7959393; doi:10.1371/journal.pone.0248459)
Supplement: Attachment — Submitted filename: review_plos_one_second_round.docx [file pone.0248459.s004.docx]

Dear Mária Potterf,

Thank you again for your valuable comments. According to your suggestions, we revised the results section. Please see the specific comments addressed in the table below.

| The manuscript now nicely and clearly specifies the advances of the current research, and how it addresses ongoing research gaps (L87-89, 102-105, etc.).Methods section was completed and now assures reproducibility. Well done! For Results sections, I list some more suggestions about how further improve data visualizations. I suggest removing multiple plots, as they do not show a clear message and therefore are difficult to interpret. E.g. from a reader perspective, I do not see why only selected ‘dates’ were plotted, and not the others (was that because of highest R squared/random selection/ something else)? As those Figs. do not show very clear message besides that they are different, I suggest removing them to keep the manuscript concise. Please see details for further information, and consider implementing them in the manuscript. | Thank you for your kind comments.  According to your suggestions we further improved the results section and removed or rearranged some figures. There are now 10 figures in the main text. We hope that now the results section is clearer and the remaining plots are easier to interpret. Please see the detailed responses below. |
| --- | --- |
| L18-19 – I suggest formulation: “Our study aim(ed) to quantify the site and forest parameters affecting …” instead of this rather ‘conclusive’ statement, as at the beginning of Abstract you show reader what your study aimed to explore | Ok, it was corrected. |
| L72-74 , 87=89– well done! nice, clear and specific summary of previous sentences. Good example of a good writing :) | Thank you! |
| L197-204 – do I get this right that dependent variables are all individual 11 bands of Sentinel-2? Please specify. | Yes, the dependent variables are values in individual Sentinel-2 bands. 10 bands were used – as they are used in land applications (the ones with -10 and -20 meter resolution – it is described in lines 158-159).  We specified information about dependent variables in lines 198-199. |
| L217 – what is meant here by the ‘changes in reflectance’? were you predicted an absolute change in e.g. SWIR values between two dates (i.e. May 2 to May 12)? I think no as one glm was fit for each band and therefore here you report the change in adjusted R squared? Please be specific. | This description refers to what is, for example, shown in figure 3. The plots show the changes in the reflectance depending on the elevation above sea level for selected bands on different dates. For example, for Red there is a large difference between the average values of reflectance on given elevation between May 2, 7 and 12 (Fig. 3). We understood changes in reflectance as the changes in the observed and model values (red lines) between successive dates visible on subsequent figures in the row.  We also added to this sentence (line 218)*: the changes in reflectance depending on elevation, were very dynamic (...)* |
| L474 = Fig. 9 remove bold fond | OK, corrected. |
| Table 3: I like this specifications, it is clear. However, you might consider to use something like ‘correlation heat maps’ to show correlation for individual bands/over time if you wish to visualize changes in R2? e.g. http://www.sthda.com/english/wiki/ggplot2-quick-correlation-matrix-heatmap-r-software-and-data-visualization This is just a suggestion for the future reference.  This could replace Fig. 3 which I suggest to remove or move to Supp. material | Thank you for your suggestion. Indeed, the correlation heat maps are a great way to visualize R2 or correlation coefficients. However, it would be difficult to produce one clear correlation plot in our case, as we have different bands as dependent variables, in different dates and for three different species. Therefore, in our opinion Table is more appropriate in this case.  In our opinion scatterplots in Fig. 3 show the dynamic differences of broadleaved trees at the beginning and the end of the growing season, and therefore should stay in the main text. However, if you and editor insist, we can move it into the supporting information. |
| Tab. 1 = Later in the text it reads that understory can contain fir as well. So, does 1 = 100% of fir trees. Are oak and beech considered therefore in one class? (broadleaved?). Please clarify. | Thank you for your comment. In general, in the GAM modelling only broadleaved/conifer understory share was taken as the predictor variable. Therefore in the Table 1 we only have left information about coniferous/broadleaved understory.  In this specific case of Scots pine, the understory species was diversified so it was possible to depict the differences in the spectral signatures. So additionally, for the purpose of visualization, we also used dominant species in case of Scots Pine stands understory.  We added that information in the Table 1. |
| Fig.4: Use the same y limits to allow visual comparison between different dates. However, I think this image is very hard to interpret, and keep in mind how curves are shifting over time. I suggest to remove it or consider alternative ways of display. From my interpretation, all % understory follows the same pattern, but there is difference mostly in SWIR1 values. Consider choosing only extreme 0&50&100% understorey values to show SWIR1 values over time? x = time, y = reflectance, group = broadleaved understory. Would that tell your story better? | Thank you for your comment. According to your suggestion, to improve interpretability, we produced new figure. It shows the reflectance variations depending on the broadleaved understory share only in SWIR1 band in selected dates during the season. However, the figure still consists the 10 different levels of broadleaved understory, but, in our opinion is now easier to visually compare. |
| Fig. 6: also, I consider this unnecessary: not clear what is the main message of the plot, ale not clear why only NIR2 band values are shown and only on one date? The story would be very different if y-axis limits were the same. I like the interpretation thought: there is difference in reflectance for stands 0-50 and >50 but this could be likely visualized simply by boxplots for groups and tested. I suggest to remove Fig.6. | Thank you for your comment. Indeed, the figure was unclear and hard to interpret, therefore we replaced it with the new one. In new figure, the mean values for the 10-year age classes is presented in one plot. It clearly shows that the stands up to 50 years old are entirely separable in the region from RE2 to NIR2. |
| Fig. 8. Again, this plot is very difficult to interpret and see differences there. Crown closure should be a discrete variable (Table 1); here is instead plot as continuous. How would the story look like for the plot would be faceted by groups (~ crown closure)? Again, another dates are missing and it is unclear why only July 1st was selected? | Thank you for your comment, we removed this figure from the text. |
| Fig. 9 = also Fir was considered as understory dominant species?? Missing from Tab .1 ? Unclear. Plot 9 is not necessary. | Thank you for your comment, as described above in the GAM modelling only broadleaved/conifer understory share was taken as the predictor variable, but in the case of Scots pine it was possible to visualize differences in three different understory species (we added that information in the Table 1. )  Furthermore, we replaced this figure analogously to Fig. 4, showing understory in case of beech stands. Also here, we provided mean values only for the SWIR1 band during the year. However, if you still think it is not necessary we could remove it. |
| Fig, 12 Very difficult to interpret. Again, there are 4 crown closure groups, but only one smooth line, difficult to spot the differences. I suggest to remove. | Thank you for your comment. We agree that this figure was hard to interpret and not very informative, therefore we removed it. |
